# Surgery and the kings of medical science: Invited Commentary: “Publication activity of chief and senior general/visceral surgeons in German university hospitals — a ten-year analysis”, Böckmann et al.

**DOI:** 10.1007/s00423-021-02242-5

**Published:** 2021-06-22

**Authors:** Ihsan Ekin Demir, Güralp O. Ceyhan, Helmut Friess

**Affiliations:** 1 Department of Surgery, Klinikum Rechts Der Isar, School of Medicine, Technical University of Munich, Munich, Germany; 2 Department of General Surgery, HPB-Unit, School of Medicine, Acibadem Mehmet Ali Aydinlar University, Istanbul, Turkey; 3 German Cancer Consortium (DKTK), Partner Site Munich, Munich, Germany

## Abstract

**Background:**

Surgeons are frequently compared in terms of their publication activity to members of other disciplines who publish in journals with naturally higher impact factors. The time intensity of daily clinical duties in surgery is yet not comparable to that of these competitor disciplines.

**Purpose:**

Here, we aimed to critically comment on ways for improving the academic productivity of university surgerons.

**Conclusions:**

To ensure high-quality science in surgery, it is imperative that surgeons actively ask for and generate the time for high-quality research. This necessitates coordinated and combined efforts of leading university surgeons at the political level and effective presentation of the magnificent studies performed by young and talented university surgeons.

Over the last century, surgery has evolved from being a pure, experience-driven handcraft to a medical discipline increasingly guided by scientific evidence. Understanding the mechanisms behind healing imbued by surgical interventions has contributed towards improvement of surgical technique and introduction of surgical innovations. It is today undoubted that future surgical excellence and best surgical outcomes will only be possible through perfect integration of emerging scientific technologies into surgical research.

The surgeon as a scientist will, therefore, lead the course of future surgical innovation.

From this perspective, the scientific activity of surgeons at the highest level of academia, i.e. at university surgical departments, is decisive for shaping the future of surgery as a science. For the same reason, the present paper by Böckmann et al. is of paramount importance for understanding the dynamics behind the scientific and publication activity of general surgeons in German university hospitals.

The present study clearly showed that the majority of the publication activity was very much concentrated on four, that is, on a very limited number, of departments, which published as much as the last 20 departments together. The main number of publications per department was between 19.6 and 7.6 in the first versus last quartile. The main cumulative impact factor in the first quartile was as high as 57.9, when compared to only 17.1 in the last quartile. As high as 21.6 of all general surgeons in these academic centres were not active as first or last authors. The publishing activity was completely independent of the size of the department. The authors correctly concluded that there are as yet unknown factors responsible for major differences in the publication activity of the departments.

The paper has high merit not only because it is one of the few studies in the field that addressed the academic activity of general surgeons at German University hospitals [1, 2]. Especially with its very last sentence in the conclusion, it draws attention to an issue that needs to be immediately improved. Indeed, we have to interrogate the reasons behind the high variance in the publishing activity of different German university surgical departments.

Certainly, the publishing activity is not the sole parameter for assessing the scientific activity of academicians. Today, performance and publishing of a high-level study take even more time and resources than in the past. “It is quality, and not quantity, that counts”, is valid more than ever, and the number of publications is not a reliable parameter for assessing scientific activity when considered on its own. Impact factor is a good indicator for the quality of science, but it also has its downsides as it does not predict the future impact and the ultimate translation of a study. Other parameters like the citation index for individual studies can be helpful for having a more accurate estimation of the publication and scientific activity.

Even after considering an improved set of evaluation parameters for assessing the scientific activity of German university surgeons, it is in our view very likely that the observations and conclusions will be very similar. The causes for the differences in the academic activity of the surgical departments are likely to be multifactorial. However, when looking at the big picture, it is conceivable that the problem can be broken down into very few key issues.

There might certainly be some geographic advantages of departments localized in leading universities of the nation with a vast scientific network and culture. The infrastructure and equipment may also not be as good for being equally competitive. However, looking at the geographically distinct locations of the four leading institutions in the top list in the Table 1 of the paper, the possibilities and conditions in the particular universities do not seem to explain the leading position of these departments. There is also no objective reason for assuming any kind of ease in grant approval or recruitment of research funds between different universities solely due to the reputation of various departments.

In our view, when it comes to explaining the difference between the individual departments, the key reason probably lies in the mentality as promoted and energetically pushed by the mentors and attending physicians of the departments. These people, who have an extraordinary amount of responsibility in the German visceral surgery department structure, should not only actively ask for scientific activity, but also put major effort into generating the best possible conditions for the young surgeons for carrying out such academic activity. Although it is possible that some departments have less difficulty in recruiting the best talents after medical university education, talent itself will be never enough for achieving the highest scientific output. It is a combination of the present scientific mentality and tradition, scientific atmosphere on the campus, the scientific network of the department, and the motivation infused by the mentors and leading surgeons of the individual departments that will eventually yield high quality science and scientific output.

As surgeons, we are frequently compared to members of other disciplines who publish in journals with naturally higher impact factors. The time intensity of our daily clinical duties is yet not comparable to that of these competitor disciplines. To ensure high-quality science in our field, it is imperative that we actively ask for and generate the time for high-quality research. It is a surgeon who needs it most to have more free time for research than members of any other medical discipline. Our access to human organs, anatomy, and physiology is unique and envied by other disciplines. We are not sure whether we are leveraging this privileged access effectively enough for advancing not only the surgical science, but also science as a whole. This necessitates coordinated and combined efforts of leading university surgeons at the political level and effective presentation of the magnificent studies performed by young and talented German university general surgeons. We are all convinced that surgery is the “king of medical disciplines”. This metaphor can be probably traced back to the unique healing abilities of the surgeons and their intellectual considerations prior to, during and after surgery, in combination with their technical excellence. Members of this discipline who exert this excellence on a daily basis, have undoubtedly the full capacity to also perform the highest level of science. It is the responsibility of all leading surgeons in the highest positions not only in Germany, but also worldwide to canalize this medical, intellectual and technical excellence into scientific activity. With dedicated minds behind such an initiative, we are convinced that academic surgeons can also become the kings of science.
